# A case of atraumatic tetanus developed initially with worsening headache in a woman regularly cared for chronic headache at an outpatient clinic

**DOI:** 10.1002/ccr3.2024

**Published:** 2019-01-31

**Authors:** Masanobu Tobinaga, Yuji Suzuki, Takashi Nakajima

**Affiliations:** ^1^ Department of Neurology Niigata National Hospital, National Hospital Organization Kashiwazaki, Niigata Japan; ^2^ Department of Neurology Brain Research Institute, Niigata University Graduate School of Medicine Niigata Japan; ^3^ Department of Pediatrics Niigata National Hospital, National Hospital Organization Niigata Japan; ^4^ Center for Integrated Human Science Brain Research Institute, University of Niigata Niigata Japan

**Keywords:** cephalic tetanus, GABA, headache, tetanospasmin, tetanus without trauma

## Abstract

We presented a case of atraumatic tetanus developed initially with severe headache. Headache may be a clue to the presence of tetanus. Clinicians who usually treat headache should consider the possibility of tetanus in patients who present with symptoms that are severe and atypical for a given patient.

## 1 INTRODUCTION

A 42‐year‐old woman who was regularly cared for chronic headache developed a severe headache 1 day after working in rice paddy field. She developed trismus afterward and was diagnosed with tetanus. Treatment was started, and her headache improved thereafter. Headache may be a clue to the presence of tetanus.

Tetanus is caused by the invasion of Clostridium tetani spores from a wound site, with tetanus prevention usually started at the time of trauma, precluding the need for diagnosis.^1^ However, 21.7%‐26% of all cases of tetanus are not accompanied by trauma, and in these cases, tetanus must be diagnosed and treated from the onset of initial symptoms.^2^, ^3^ Trismus is a pathognomonic symptom in tetanus, appearing in more than half of all cases,^5^ and its presence makes diagnosis easier. Cases with rare symptoms are much more difficult to diagnose, and this may delay the start of treatment. Despite being listed as a symptom of tetanus, headache is a rare initial symptom. We report the case of a woman with atraumatic tetanus who presented with severe headache as the initial symptom; we could diagnose the disease by trismus developed during the clinical course.

## 2 CASE PRESENTATION

The patient was a 42‐year‐old woman. She had suffered from migraine and tension‐type headaches since her twenties. The migraine headache was described as pulsatile, bilateral, and on the forehead, persisting from a few hours to half a day. It occurred seven to eight times a month irrespective of menstruation and was accompanied by aura (partial deficit of the left visual field lasted approximately 10 minutes), light sensitivity, and nausea. She took oral loxoprofen 60 mg to treat the headache, on average, <15 days a month, which did not meet the standard of mediation‐overuse headache. The tension‐type headache was followed by muscle stiffness from the shoulders to the neck and was exacerbated by fatigue. The frequency of pain attacks was one per week. The duration was 1 or 2 days. The headache was bilaterally located, of pressing quality, was not aggravated by walking, not associated with nausea and photophobia.

Eight days before admission, the patient had engaged in farm work. During this work, she reported that grass fragments had entered her right eye while operating a mower. She experienced strong pain and a foreign body sensation but stated that there had been no bleeding or inflammation. The next morning, she reported general malaise and a persistent pulsatile headache on both sides of her forehead, accompanied by a fever of 38.5°C by the evening. The headache was accompanied by nausea and occasional vomiting; it was aggravated by turning her face downward and was not associated with photophobia and phonophobia. The effect of loxoprofen was inadequate and lasted only a few hours. The symptoms gradually worsened over the following 3 days, and the nature of the headache changed to a pain that tightened around the whole head. Nausea appeared in addition to the headache, so she presented to a nearby clinic. Head computed tomography was performed and showed no evidence of cerebral hemorrhage. She was discharged with reassurance; however, her headache gradually worsened and she consulted the clinic again 2 days later and was referred to our hospital with suspected meningitis.

**Figure 1:**
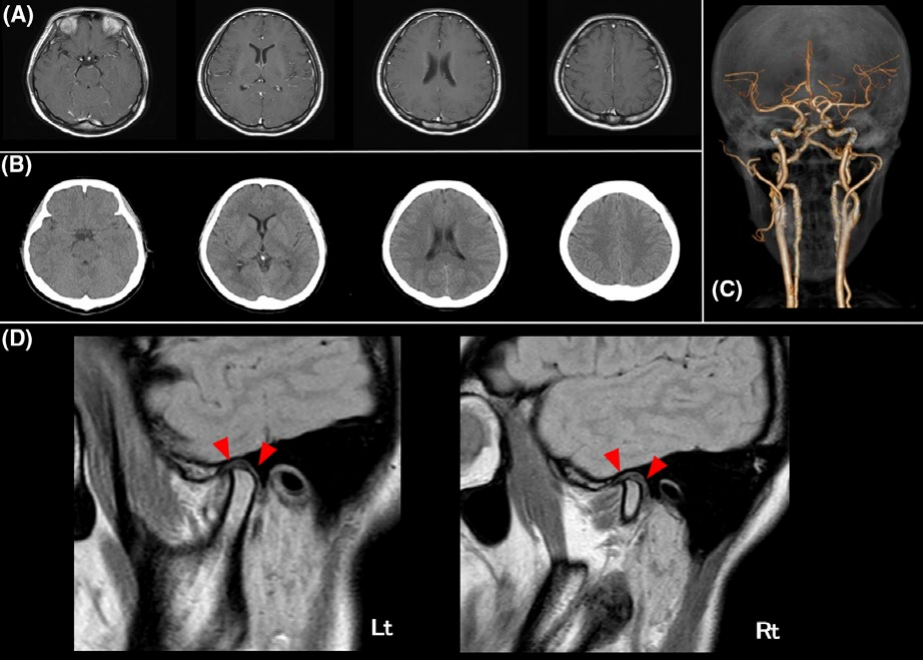
A, Contrast‐enhanced MRI showed no contrast effect in the meninges or brain parenchyma. B, Head computed tomography showed no cerebral hemorrhage. C, Computed tomography angiography did not reveal vertebral artery dissection or cerebral aneurysm. D, Proton density weighted MRI showed a narrow temporomandibular joint head and thinning of the temporomandibular disk (red arrow head) but did not reveal a temporomandibular disorder

Neurological examination, laboratory data from blood and spinal fluid (Table 1), and contrast‐enhanced head magnetic resonance imaging (Figure 1A) showed neither meningitis nor any other abnormality that could explain the headache. The serum antibody of tsutsugamushi disease, which is a kind of Lyme disease, was negative. Systemic reactions including BHL, serum Ca high values, which suggest sarcoidosis, were negative. Head computed tomography (Figure 1B) and computed tomography angiography (Figure 1C) also revealed no cerebral hemorrhage, vertebral artery dissection, or cerebral aneurysm. At this time, she described the headache as 10/10 on a numeric rating scale (NRS). Intravenous infusion of 1000 mg acetaminophen over 2 days reduced the severity of the headache to an NRS of five. Although the patient reported a considerable improvement in the headache, she stated that the mild occipital pain remained. A stinging pain was described that lasted for several minutes and was mixed with a constant and background occipital pain. We considered occipital neuralgia at this point and started treatment with 400 mg of oral carbamazepine, which improved the headache to an NRS of two by the following day.

**Table 1:** Initial laboratory data from blood and spinal fluid

**Blood test**			
Total bilirubin	1.2 mg/dL	Rheumatoid factor	4.0 IU/mL
Aspartate		White blood cell	3300/µL
Aminotransferase	169 IU/L	Red blood cell	5 390 000/µL
Alanine		Hemoglobin	15.5 g/dL
Aminotransferase	310 IU/L	Hematocrit	45.0%
Lactate dehydrogenase	374 IU/L	Platelets	145 000/µL
Alkaline phosphatase	195 IU/L	Basophil	0.3%
Gamma‐glutamyl		Eosinophil	0.0%
Transpeptidase	59 IU/L	Neutrophil	74.8%
Creatine kinase	37 IU/L	Lymphocyte	17.0%
Blood urea nitrogen	22.0 mg/dL	Monocyte	7.9%
Creatinine	0.78 mg/dL	Prothrombin time	12.7 s
Na	140 mEq/L	Prothrombin time	
K	3.0 mEq/L	International normalized ratio	1.09
Cl	97 mEq/L	Activated partial thromboplastin	
Ca	9.1 mg/dL	Time	31.0 s
IP	1.7 mg/dL	D‐dimer	510 ng/mL
Blood sugar	113 mg/dL	Rickettsia tsutsugamushi	
Hemoglobin A1C	5.0%	Antibody	Negative
C‐reactive protein	6.3 mg/dL	Blood Culture	Negative
**CSF analysis**			
Color	Clear	Total protein	36 mg/dL
Specific weight	1.006	Glucose	60 mg/dL
Cell count	6 cells/mm^3^	Cl	117 mg/dL
Neutrophil	1 cells/mm^3^	HSV antibody	Negative
Monocyte	5 cells/mm^3^	Cerebrospinal fluid culture	Negative

Blood tests showed liver dysfunction, inflammatory responses, and leukocytopenia. Cerebrospinal fluid test showed no evidence of meningitis.

On the fifth day of admission, the patient reported difficulty in opening her mouth. The distance between the upper and lower incisors was 5 mm. Temporomandibular joint MRI showed no abnormality and excluded temporomandibular joint disease (Figure 1D). Based on the history and characteristic symptom, she was diagnosed with tetanus and treatment was started with tetanus toxoid vaccine, human tetanus immunoglobulin (3000 units), and penicillin G (12 million units). By the next day, this treatment had improved the remaining headache that encircled the whole head to an NRS of 0, but an occipital headache remained.

**Figure 2:**
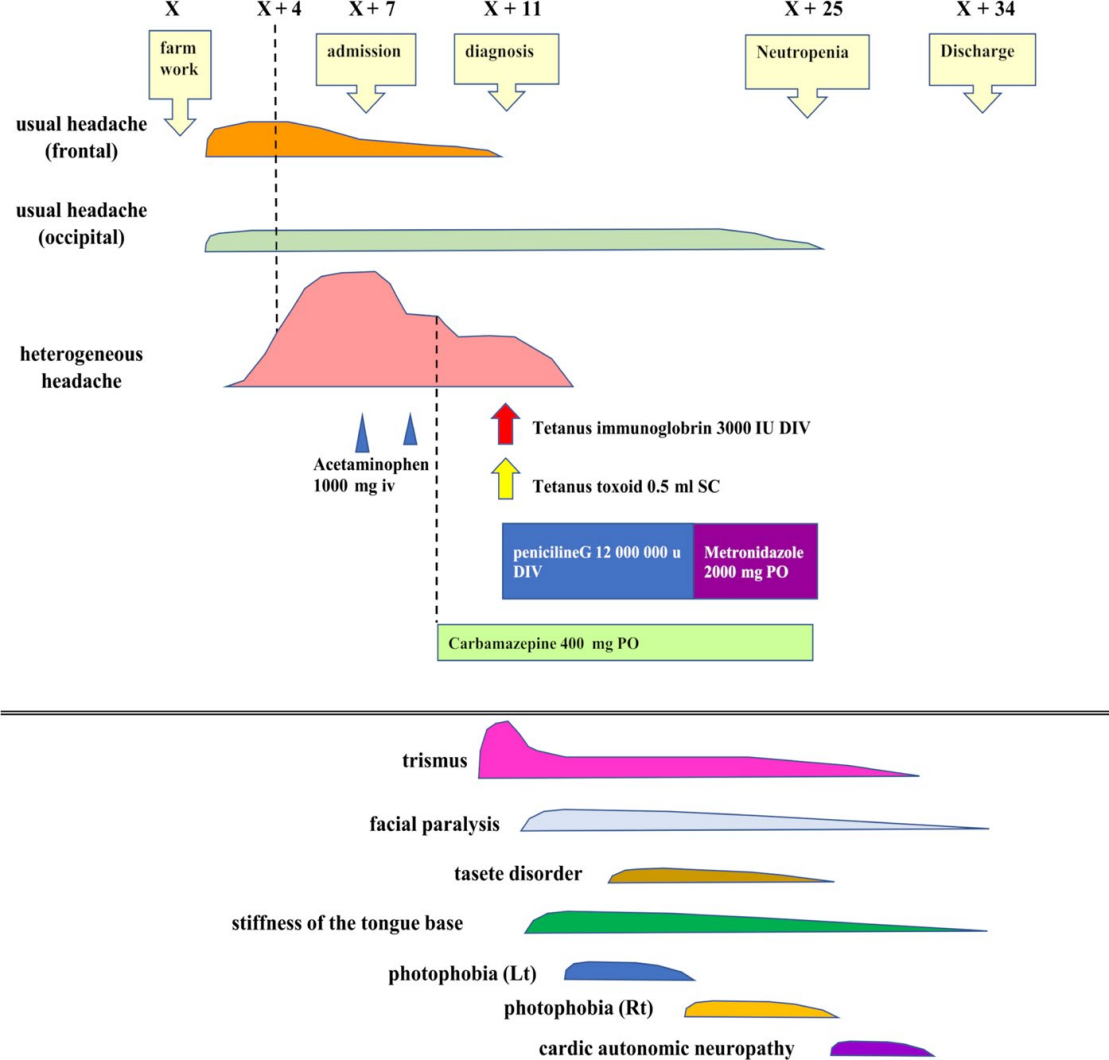
Clinical course and treatment. Heterogeneous headache (NRS 7), characterized by pain that constricted the whole head, developed on day 4 after the farm work; the headache worsened the next day (NRS 10). Starting with trismus 10 days after the headache, various other symptoms of tetanus then appeared. Abbreviations: iv, intravenous; Lt, left; NRS, numeric rating scale; PO, per os; Rt, right; x, index event

During the subsequent disease course, the patient developed various symptoms, including facial nerve paralysis, stiffness of the tongue base, photophobia, and cardiac autonomic nervous disorder (Figure 2). She developed facial nerve palsy and stiffness of the tongue base the day after the appearance of trismus. The facial nerve palsy was bilateral and peripheral. Her nasolabial grooves were equal on the both sides, but the weakness of the orbicularis oculi muscle was left‐side dominant, which caused leakage from the corner of her mouth when she took fluid orally. Although the stiffness of the tongue base caused a sensation of throat obstruction, she could breathe and swallow normally. She developed dysarthria due to the trismus and weakness of orbicularis oris muscle. Photophobia appeared in the order of the left to the right side, and she experienced a loss of taste.

Although most symptoms began to improve with treatment, she reported palpitations under mild exertion on the 20th day. The coefficient of variation of the R‐R intervals on an electrocardiogram was 1.71% at this point, indicating autonomic dysfunction, but this improved to within the normal range 1 week later (3.14%). She was followed as an outpatient, and after 7 weeks she had regained full strength of the orbicularis oris muscle and her persistent occipital pain had improved.

## 3 DISCUSSION

Our case was not accompanied by trauma; hence, it was not possible to isolate and identify Clostridium tetani from the wound site, and subsequent culture tests of the blood and spinal fluid were negative. It is usually difficult to identify Clostridium tetani^1^; therefore, in this case, the treatment for tetanus was started when trismus was observed, after negating temporomandibular disorder. Subsequently, flaccid cranial nerve palsy appeared and was improved after the treatment for tetanus; hence, this case was diagnosed as cephalic tetanus from its clinical course.

Despite being listed as a potential symptom, headache is a rare initial presentation of tetanus. Furthermore, our case was complicated by the fact that the patient had a history of tension headaches and migraine. Thus, despite extensive investigation, appropriate treatment was not started until the appearance of trismus.

Although the patient had an established history of migraine and tension headache, it was apparent that the presenting headache had a different characteristic to normal. The patient had never experienced a headache of such severity or that involved tightening of the whole head with either headache in the past. These atypical symptoms are typical of tetanus and are thought to be due to tetanospasmin produced by Clostridium tetani. The mechanism of action is thought to be the inhibition of GABAergic nerves by tetanospasmin.^1^, ^3^, ^6^, ^7^ Appropriate treatment for tetanus ameliorated the headache, which is consistent with this hypothesis.

In this case, symptoms were confined to the head, including cranial nerves VII, XI, and XII, so a diagnosis of cephalic tetanus was made. In several reported cases of cephalic tetanus, pain was the primary symptom (eg, headache, facial pain, and sore throat).^8^ Thus, to ensure the early diagnosis of cephalic tetanus, careful attention should be paid to severe headaches as an initial symptom.

## 4 CONCLUSION

In cases of tetanus without apparent trauma, particularly cephalic tetanus, headache may be a clue to the presence of tetanus. Clinicians who usually treat headache should consider the possibility of tetanus in patients who present with symptoms that are severe and atypical for a given patient.

## CONFLICT OF INTEREST

None declared.

## AUTHOR CONTRIBUTION

MT: is the primary physician, made clinical diagnosis, and wrote manuscript draft. YS: was the corresponding author, reviewed, revised, and supervised drafting of the manuscript. TN: revised the manuscript and finally approved the version to be published.

## References

[1] Bleck TP . Tetanus: pathophysiology, management, and prophylaxis. Dis Mon. 1991;37:545‐603. 1874121 10.1016/0011-5029(91)90008-y

[2] Tomoda Y , Kagawa S , Kurata S , Nakatake N , Tanaka K . Tetanus without apparent history of trauma. J Gen Fam Med. 2018;19:61‐62. 29600132 10.1002/jgf2.153 PMC5867140

[3] Farrar JJ , Yen LM , Cook T , et al. Tetanus. J Neurol Neurosurg Psychiatry. 2000;69:292‐301. 10945801 10.1136/jnnp.69.3.292 PMC1737078

[4] Marulappa VG , Manjunath R , Mahesh N , Maligegowda L . A ten year retrospective study on adult tetanus at the epidemic disease (ED) hospital, Mysore in Southern India: a review of 512 cases. J Clin Diagn Res. 2012;6:1377‐1380. 23205351 10.7860/JCDR/2012/4137.2363 PMC3471487

[5] Giannini L , Maccari A , Chiesa V , Canevini MP . Trismus, the first symptom in a challenging diagnosis of Tetanus. BMJ Case Rep. 2016;2016:2015213897. 10.1136/bcr-2015-213897 PMC5483546 26869628

[6] Mellanby J , Green J . How does tetanus toxin act? Neuroscience. 1981;6:281‐300. 6164010 10.1016/0306-4522(81)90123-8

[7] Ohashi N , Sasaki M , Ohashi M , Kamiya Y , Baba H , Kohno T . Tranexamic acid evokes pain by modulating neuronal excitability in the spinal dorsal horn. Sci Rep. 2015;5:13458. 26293582 10.1038/srep13458 PMC4544020

[8] Burgess JA , Wambaugh GW , Koczarski MJ . Report of case: reviewing cephalic tetanus. J Am Dent Assoc. 1992;123:67‐70. 10.14219/jada.archive.1992.0170 1619169

